# Analysis of the Piezoelectric Properties of Aligned Multi-Walled Carbon Nanotubes

**DOI:** 10.3390/nano11112912

**Published:** 2021-10-30

**Authors:** Marina V. Il’ina, Oleg I. Il’in, Nikolay N. Rudyk, Olga I. Osotova, Alexander A. Fedotov, Oleg A. Ageev

**Affiliations:** 1 Institute of Nanotechnologies, Electronics and Electronic Equipment Engineering, Southern Federal University, 347922 Taganrog, Russia; aafedotov@sfedu.ru (A.A.F.); ageev@sfedu.ru (O.A.A.); 2 Research Laboratory of Functional Nanomaterials Technology, Southern Federal University, 347922 Taganrog, Russia; oiilin@sfedu.ru (O.I.I.); nnrudyk@sfedu.ru (N.N.R.); osotova@sfedu.ru (O.I.O.); 3 Research and Education Center “Nanotechnologies”, Southern Federal University, 347922 Taganrog, Russia

**Keywords:** carbon nanotube, piezoelectric, nanogenerator, atomic force microscopy, nanoelectromechanical systems, NEMS, nanopiezotronics

## Abstract

Recent studies reveal that carbon nanostructures show anomalous piezoelectric properties when the central symmetry of their structure is violated. Particular focus is given to carbon nanotubes (CNTs) with initial significant curvature of the graphene sheet surface, which leads to an asymmetric redistribution of the electron density. This paper presents the results of studies on the piezoelectric properties of aligned multi-walled CNTs. An original technique for evaluating the effective piezoelectric coefficient of CNTs is presented. For the first time, in this study, we investigate the influence of the growth temperature and thickness of the catalytic Ni layer on the value of the piezoelectric coefficient of CNTs. We establish the relationship between the effective piezoelectric coefficient of CNTs and their defectiveness and diameter, which determines the curvature of the graphene sheet surface. The calculated values of the effective piezoelectric coefficient of CNTs are shown to be between 0.019 and 0.413 C/m^2^, depending on the degree of their defectiveness and diameter.

## 1. Introduction

Recent advances in nanotechnology have generated increased interest in studying the electromechanical properties of nanoscale materials that do not exhibit piezoelectric properties in bulk [1,2,3,4,5,6,7]. The source of anomalous piezoelectric properties in non-piezoelectric materials is the violation of the structural symmetry on the surfaces and interfaces [8,9,10,11,12,13,14]. Due to high mechanical strength and elasticity, much attention in this direction has been attracted by carbon nanostructures subjected to various modifications [2,15,16,17,18,19,20,21]. It has been experimentally and theoretically proven that a graphene sheet demonstrates surface piezoelectricity and flexoelectricity when non-centrosymmetric pores are formed in it [19,22], when creating a bending moment and biaxial strain [9,15,17], and through the selective surface adsorption of atoms [2,16,19,23]. Through this connection, we can observe an active use of the flexo- and piezoelectric properties of carbon nanostructures in order to create promising elements of nanoelectromechanical systems (nanogenerators, sensors, actuators, etc.) [15,24,25,26,27,28].

From a technological point of view, the simplest way to create piezoelectricity in a graphene sheet is to form a bending moment, since this does not require additional surface modification. In this case, it is interesting that a significant bending moment is initially formed in carbon nanotubes (CNTs), which can lead to the manifestation of the surface piezoelectricity [22]. For the first time, we discovered the anomalous piezoelectric properties of CNTs via experimental research studying the memristor effect in a deformed CNT [29,30,31]. The appearance of the internal electric field strength in a deformed carbon nanotube connected with its piezoelectric properties was shown [29,31]. Using atomic force microscopy, we demonstrated the piezoelectric response of multi-walled CNTs [31,32]. The current generated upon deformation of an individual CNT reached −24 nA. The surface potential at the tops of the deformed CNTs ranged from 268 to −110 mV, depending on the type and magnitude of the deformation [32].

This paper presents the results of studies of the structure and piezoelectric properties of multi-walled carbon nanotubes grown by plasma chemical vapor deposition (PECVD). We present an original technique for assessing the piezoelectric coefficient of CNTs based on the analysis of the internal electric field arising in CNTs under controlled longitudinal deformation. The piezoelectric coefficients of CNTs are experimentally determined for the first time. The influence of the growth temperature and the thickness of the catalytic Ni layer on the structure of CNTs and the value of their piezoelectric coefficient are studied. The relationship between the piezoelectric coefficient of CNTs and their defectiveness is established, and its correlation with the diameter of the CNT and the curvature of the surface of the graphene sheet forming it is studied. A comparative study of the obtained values of the piezoelectric coefficients of CNTs and the values of the main nanoscale piezoelectric materials is presented.

## 2. Materials and Methods

To establish the influence of the growth temperature and the thickness of the catalytic Ni layer on the structure of CNTs and the magnitude of its piezoelectric response, 15 arrays of aligned carbon nanotubes were fabricated and studied. A chemically purified silicon wafer Si (100) (GIRMET Itd., Moscow, Russia) was used as a substrate when creating experimental samples. A barrier (TiN) layer of 100 nm thickness and a catalytic (Ni) layer with a thickness of 5 to 30 nm were formed on the substrate by magnetron sputtering. Aligned CNTs were grown by the plasma-enhanced chemical vapor deposition (PECVD) method. The formation of catalytic Ni centers from a continuous film was carried out by heating the samples to a temperature ranging from 615 to 690 °C for 20 min in an atmosphere of argon (40 sccm) (Voessen, Moscow, Russia) and ammonia (15 sccm) (Voessen, Moscow, Russia) at a pressure of 4.5 Torr. After that, the substrates were exposed to ammonia plasma (210 sccm, 15 W) for 1 min. Plasma was initiated using a high-voltage direct current source. Then, acetylene (70 sccm) (Voessen, Moscow, Russia) was introduced into the chamber simultaneously with ammonia, and the vertically aligned carbon nanotubes were grown with the use of the “tip” mechanism for 15 min at a temperature ranging from 615 to 690 °C and a plasma power of 40 W.

**Figure 1:**
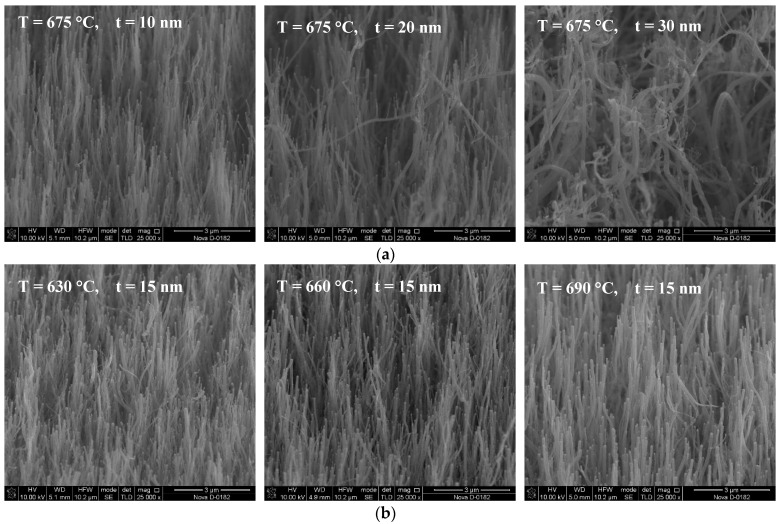
(**a**) SEM images of CNT arrays grown at a temperature of 675 °C and a catalytic layer thickness *t* ranging from 10 to 30 nm, and (**b**) at a catalytic layer thickness of 15 nm and an increase in temperature from 660 to 690 °C.

The geometrical parameters of the grown CNT arrays were measured using the Nova NanoLab 600 (FEI, Eindhoven, Netherlands) scanning electron microscope (SEM). Figure 1 shows SEM images of some of the CNT arrays.

The structural analysis of the experimental samples was conducted via transmission electron microscopy (TEM) using the Tecnai Osiris (FEI, Eindhoven, Netherlands), and also through the use of the Renishaw InVia Reflex (Renishaw plc, Wotton-under-Edge, UK) Raman spectrometer with laser excitation wavelengths of 514 nm. The study of current–voltage characteristics was carried out in the current spectroscopy mode of scanning tunneling microscopy (STM) using the Ntegra (NT-MDT, Zelenograd, Russia) probe nanolaboratory (PNL). The duration and amplitude of the sawtooth voltage pulses were 1 s and 3 V, respectively. The current limit was set to 50 nA to protect the measuring system and to prevent the destruction of the CNT due to high-density current flow in the STM system.

The study of the surface potential of the CNT arrays was carried out using the two-pass method of the Kelvin probe of atomic force microscopy (AFM) (NT-MDT, Zelenograd, Russia) at a distance of 12 nm between the AFM probe and the CNTs surface. A map of the distribution of the current generated by aligned CNTs during their deformation by the AFM probe was obtained using the Hybrid mode of AFM, which is represented a set of rapid measurements of force curves with processing of the current response in real time.

## 3. Results and Discussion

Analysis of the SEM images of the CNT arrays showed that an increase in the thickness of the catalytic Ni layer to 20 nm and more led to an increase in the disorientation and hierarchical structure of the CNT arrays (Figure 1). An increase in the growth temperature from 615 to 690 °C did not lead to significant changes in the structure of the array as a whole, but affected the geometric parameters of CNTs (Figure 1).

**Figure 2:**
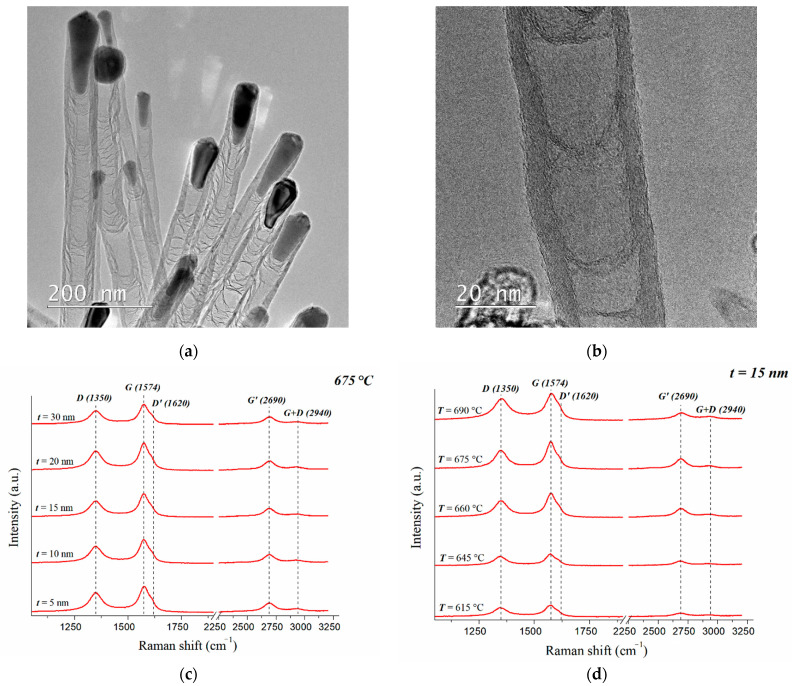
TEM images of the studied CNTs (**a**,**b**). Raman spectra of CNT arrays grown at a temperature of 675 °C and a catalytic layer thickness of 5 to 30 nm (**c**), and at a catalytic layer thickness of 15 nm with an increase in temperature from 615 to 690 °C (**d**).

The analysis of the studied arrays using TEM showed that all the CNTs were multi-walled and had bamboo-like defects (Figure 2a,b). The analysis conducted using Raman spectroscopy also showed that the obtained spectra corresponded to the spectra of multi-walled carbon nanotubes (Figure 2c,d) [33,34,35,36]. The RBM mode, which is typical for single-walled CNTs, was absent. The full width at half maximum (FWHM) of the G^−^ peak was quite large in the range of 58 to 71 cm^−1^. There was also no splitting of G^+^–G^−^ due to the large number of tubes inside multi-walled CNTs. The G-peaks for various samples lay in the shift range of 1569–1577 cm^−1^ and demonstrated a weak asymmetric characteristic peak of vibration in the sp^2^ plane with a maximum close to the graphite [37]. The D-peak responsible for vibrations outside the plane of the graphite structure was found at a shift range of 1347–1350 cm^−1^. The relatively high intensity of the D-peak could have been connected with a large number of walls inside the multi-walled CNT, as well as with a presence of bamboo-like defects and catalytic Ni particles in the CNT tops (Figure 2c,d). A decrease in the relative intensity of the D-peak and a decrease in FWHM from 71 to 58 cm^−1^ demonstrated an increase in the structural perfection of CNTs with the increasing of the growth temperature and the thickness of the catalytic layer (Figure 2c,d).

Furthermore, in the region of the G-peak, the presence of a D’-peak was observed in the shift range of 1620 cm^−1^, which indicated randomly distributed surface charges or impurities in the graphene layers of CNTs [38]. As a result, localized vibrational modes of surface charges or impurities could interact with the extended phonon modes of graphene, resulting in the observed splitting of G–D’ with a maximum D’ in the region of ~1620 cm^−1^ [38]. The spectrum also contained a G’-peak at a shift of ~2690 cm^−1^, which was associated with a two-phonon process of the second order and was dependent on the energy of the exciting laser, as well as a G + D peak in the shift range of ~2940 cm^−1^.

**Figure 3:**
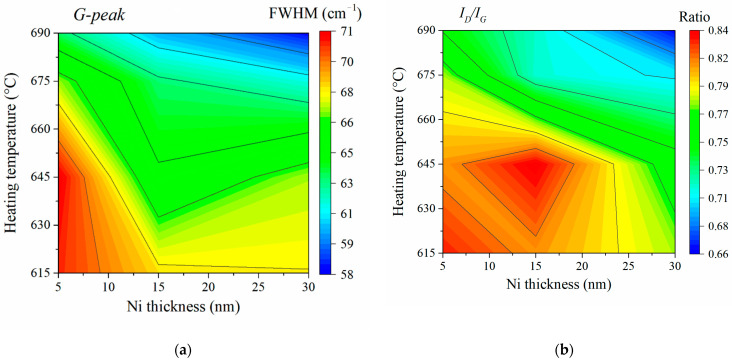
Maps of the FWHM distribution of the G-peak (**a**) and the ratio of the intensities of the D- and G- peaks I_D_ /I_G_ (**b**) of the Raman spectra of CNT arrays versus the growth temperature and the thickness of the catalytic layer.

Based on the obtained data, we designed maps of the FWHM distribution of the G-peak and the ratio of the intensities of the D- to G-peak (I_D_/I_G_) (Figure 3), which allowed us to assess the influence of the growth temperature and the thickness of the catalytic Ni layer on the defectiveness of CNTs. In Figure 3b, it can be seen that the relative intensity ratios for different images ranged from 0.66 to 0.84.

The analysis of the presented distribution maps showed that the defectiveness of CNTs decreased both with the increasing of the temperature and with the increasing of the thickness of the catalytic layer (Figure 3). The smallest defectiveness of the CNT structure (I_D_/I_G_ = 0.66, FWHM = 58 cm^−1^) was observed in the arrays grown at a temperature of 690 °C and a catalytic Ni thickness of 30 nm. The most defective CNTs turned out to be arrays (I_D_/I_G_ = 0.84, FWHM = 71 cm^−1^) grown at the minimum temperature and thickness values of the catalytic Ni layer (615 °C and 5 nm). At the same time, it should be noted that the increasing of the thickness of the catalytic layer of Ni led not only to a decrease in the defectiveness of CNTs, but also to an increase in the disorientation and hierarchy of the array as a whole (Figure 1). In addition, a shift of the G- and D-peaks towards an increasing trend was observed together with an increase in the thickness of the catalytic layer.

In addition, when comparing the FWHM of the G-peak and the average diameter (D) of CNTs (see Table 1), it can be seen that the FWHM of the G-peak increased with the decreasing of the CNT diameter. This dependence could be connected with a decrease in the CNT diameter and an increase in the surface curvature of graphene in the CNT walls, which caused additional phonon scattering and broadening of the G peak in the spectra.

**Figure 4:**
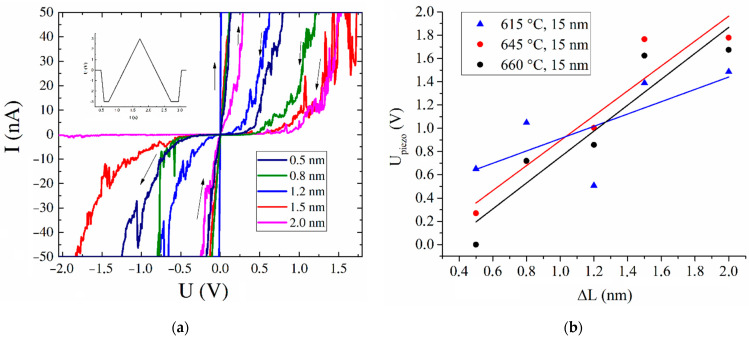
Current–voltage characteristic of CNTs grown at T = 615 °C and t = 15 nm at different tensile strain values ΔL (**a**). The CVCs were obtained by applying a sawtooth voltage pulse with an amplitude of 3 V and a duration of 1 s. Dependence of the potential difference between the top and bottom of the CNT (U_piezo_) on the value of the relative deformation of CNTs grown at different temperatures (**b**).

One of the main characteristics of material piezoelectric properties is the piezoelectric coefficient *e*, a component of the third-rank tensor that describes the linear response of electric polarization, which is a first-order tensor, to the applied strain, which is a second-rank tensor [12]. At the moment, the main method for determining the piezoelectric coefficient of nanoscale materials is piezoresponse force microscopy. However, this method is difficult in terms of studying aligned carbon nanotubes due to the mobility of their tops and their rather developed surface morphology. To determine the diagonal component of the piezoelectric coefficient of aligned CNTs, we developed an original measurement technique based on the analysis of the current–voltage characteristic (CVC) of a nanotube with controlled tensile deformation. The tensile deformation was chosen because the flexoelectric effect of a vertically aligned CNT can be neglected due to the small strain gradient (the maximum value dΔL/dx was 0.002 at ΔL = 2 nm and a CNT length of 8.3 µm) for this type of deformation. However, further in the text, we will use the term “effective piezoelectric coefficient”, since the flexo- and piezoelectric effects were inseparable during the measurement. Controlled deformation in an aligned CNT was formed under the action of an inhomogeneous electric field based on a previously developed technique [39]. As we showed earlier, in a deformed CNT, the internal electric field arises in connection with the formation of piezoelectric charges [29]. During the tensile deformation process, a positive piezoelectric charge will be concentrated at the top of the nanotube and the internal field strength will be directed opposite to the direction of the deformation [29]. Subsequent application of an external electric field to an unevenly deformed carbon nanotube will lead to a redistribution of the initial deformation and the internal electric field as a result of the manifestation of the inverse piezoelectric effect. [29,30,39,40,41]. This process was clearly reflected in the CVC of a deformed CNT in the form of a hysteresis loop, the area of which depended on the magnitude of the initial deformation and the stress applied (Figure 4).

To calculate the diagonal component of the effective piezoelectric coefficient *e*, the resistance of CNTs was calculated at different tensile deformation values ranging from 0.5 to 2.0 nm. The minimum resistance of CNTs in a low-resistance state occurs when the internal electric field is caused by the initial deformation and is completely compensated by the external electric field due to the redistribution of the CNT deformation [29]. A further increase in the resistance of CNTs in a low-resistance state is determined by the uncompensated initial deformation of the CNT and the internal electric field [29]. Thus, the minimum resistance of a CNT in a low-resistance state characterizes only the internal resistance of a CNT RCNT, which is not related to its piezoelectric properties. A change in the initial deformation of CNTs ΔL leads to an increase in the internal electric field E_def_ = U_piezo_/L and an increase in the nanotube resistance R:

(1) $$R=R_{CNT}+\frac{U_{piezo}}{I}$$

where U_piezo_ is the potential difference between the top and bottom of the CNT, resulting from the deformation of the nanotube. The value of U_piezo_ is proportional to the value of the longitudinal polarization P_||_ of the nanotube: U_piezo_ = P_||_·L/(ε_||_ · ε_0_), where ε_0_ is the dielectric constant; ε_||_ = 1 + L^2^/(24 · D^2^ ln(2L/D – 1)) is the effective longitudinal dielectric constant of the CNTs [42], and L is the length of the CNTs.

The value of the potential U_piezo_ was calculated on the basis of the Equation (1) taking into account the CVCs of the studied CNTs obtained at different deformations. The dependences of the U_piezo_ value on the deformation for CNTs grown at temperatures of 615, 645, and 660 °C and t = 15 nm are shown in Figure 4b. It is shown that the U_piezo_ value increased linearly with the increasing of the CNT tensile deformation. In turn, the U_piezo_ value was proportional to the value of the longitudinal polarization of the CNT. This dependence suggests that an increase in the polarization of the CNT occurred with the increasing of its deformation, which corresponded to the direct piezoelectric effect. The slope of the U_piezo_(ΔL/L) dependences (Figure 4b) should depend on the defectiveness and piezoelectric properties of nanotubes.

On the other hand, the magnitude of the internal electric field of a CNT is determined by its piezoelectric properties and the magnitude of the relative deformation [29] (see Supplementary Material) and value of the potential U_piezo_ is:

(2) $$U_{piezo}=E_{def}\cdot L=\frac{e}{\varepsilon_{0}\varepsilon_{\left|\right|}}\frac{\Delta L\cdot L}{L}$$

Hence, the diagonal component of the effective piezoelectric coefficient is:

(3) $$e=\frac{U_{piezo}\varepsilon_{0}\varepsilon_{\left|\right|}}{\Delta L}$$

The analysis of the CVCs for CNTs grown at T = 615 °C and t = 15 nm showed that the internal resistance of the CNT corresponded to the resistance that was observed in the low-resistance state during CNT deformation ΔL = 1.2 nm and was R_CNT_ = 0.3 MΩ (Figure 4). A further change in the deformation of CNTs led to an increase in the resistance R in a high-resistance state from 16.5 to 48.5 MΩ with an increase in deformation from 0.5 to 2 nm, respectively (Figure 4). Hence, the U_piezo_ value, taking into account Equation (1), ranged from 0.49 to 1.45 V at a fixed current equal to 30 nA. The calculation of the piezoelectric coefficient based on the Equation (3) showed that, for the studied CNTs, the effective piezoelectric coefficient *e* was 0.281 ± 0.023 C/m^2^.

It should be noted that the observed effects of resistive switching CNT are similar to the manifestation of the piezoresistive effect, which leads to a change in the resistance of a CNT under the action of deformation as a result of a change in the band gap of the nanotube [43,44,45,46]. However, it was observed in previous works that the piezoresistive effect cannot lead to the formation of surface potential on deformed CNTs [29,32], which suggests the manifestation of the piezoelectric effect in multi-walled CNTs. In addition, an analysis of the literature showed that the results of experimental studies of the conductivity of aligned multi-walled CNTs do not always agree with the piezoresistive effect theory [47,48].

**Figure 5:**
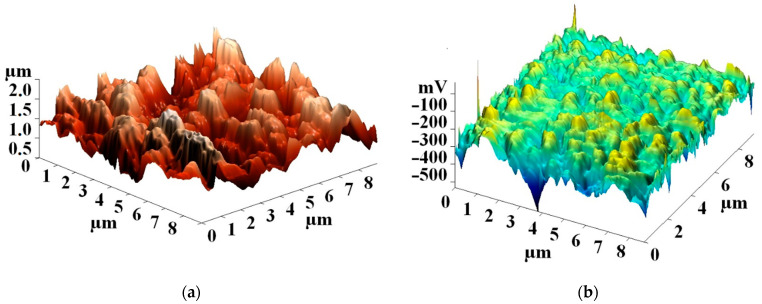
Topology (**a**) and distribution of the surface potential (**b**) of the aligned multi-walled CNTs combined into bundles.

Investigations of the surface potential of the CNT array grown at T = 615 °C and t = 15 nm using the Kelvin probe method also showed the presence of a potential of −0.6 to −0.1 V on the CNT bundles (Figure 5). The formation of the surface potential was caused by the bending deformations of the carbon nanotubes forming the bundle. The combination of individual CNTs into a bundle occurred under the action of van der Waals forces in the process of scanning the CNT array in the semicontact AFM mode [49]. The measured potential values were less than the U_piezo_ values (from 0.49 to 1.45 V), calculated taking into account Equation (1), which was probably due to the different magnitude and type of CNT deformation.

**Figure 6:**
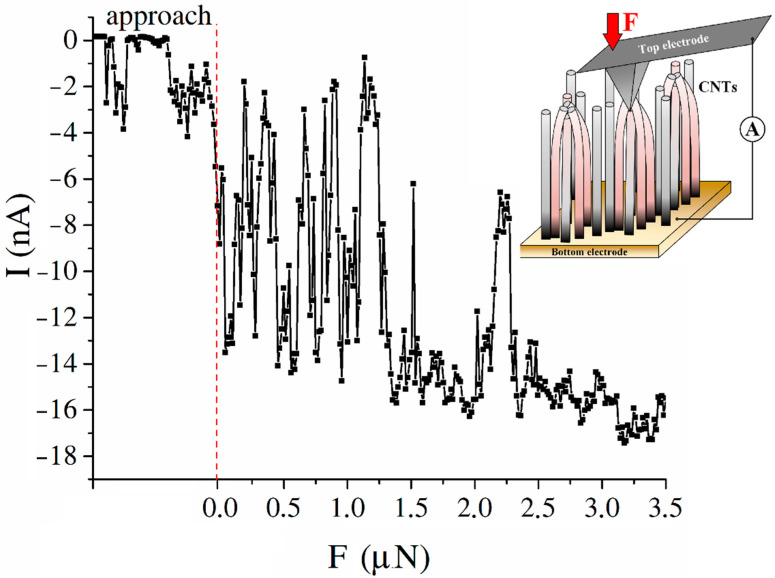
Dependence of the current generated during the deformation of CNTs grown at a temperature of 615 °C and a catalytic nickel layer thickness of 15 nm on the pressing force of the AFM probe to the CNT array surface. The inset shows a schematic representation of the measurement process.

In addition, a current was detected in the “top electrode/CNT bundle/bottom electrode” system when the AFM probe, acting as the upper electrode, approached the top of the CNT bundle (Figure 6). The current was associated with the formation of a potential difference between the CNT bundle and the grounded upper electrode. The dependence of the current detected in the “top electrode/CNT bundle/bottom electrode” system on the pressing force of the AFM probe is shown in Figure 6.

Analysis of the obtained dependence showed that a current of between 0 and −3 nA was detected in the “top electrode/CNT bundle/bottom electrode” near the CNT array surface (~100 nm) system, which was associated with the long-range effect of the CNT surface potential (Figure 6). The current increased to −8 nA at the moment of contact of the AFM probe with the CNT surface. Then, the detected current increased to 18 nA with an increase in the pressing force of the AFM probe to the CNT surface to 3.5 μN (Figure 6). In this case, current surges of up to −2 nA were observed, which were probably associated with the mobility of the CNT tops during the measurement and the change in the value of bending deformations of the CNT.

Thus, the presence of the surface potential and the corresponding current of a deformed CNT confirms our assumption that the resistive switching of the deformed CNT is associated primarily with the formation of an internal electric field as a result of the manifestation of the piezoelectric effect, rather than the piezoresistive effect.

Similar measurements to determine the effective piezoelectric coefficient of the CNT were produced for all the samples. The values of the effective piezoelectric coefficients of the CNTs are shown in Table 1.

**Figure 7:**
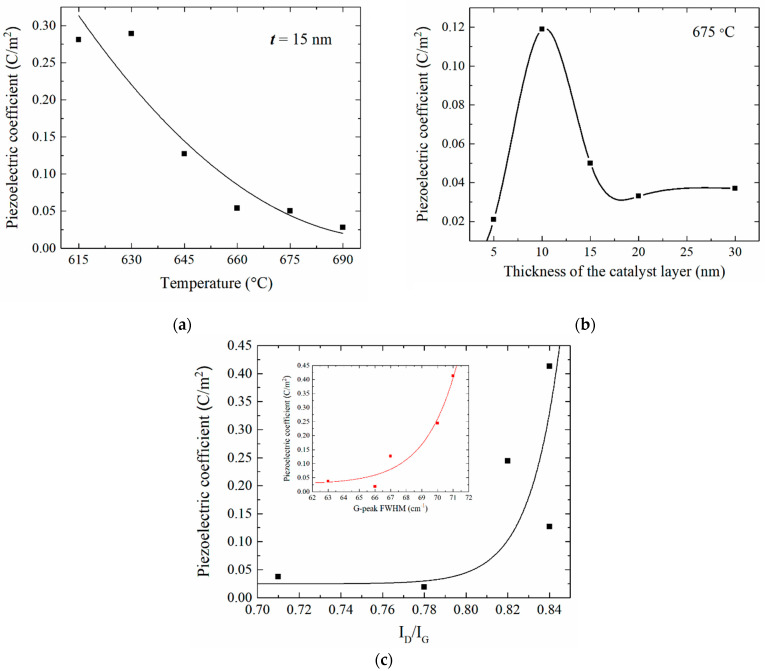
(**a**) Dependences of the effective piezoelectric coefficient of CNTs on the growth temperature at a catalytic Ni layer of 15 nm, (**b**) on the thickness of the catalytic Ni layer at a temperature of 675 °C and (**c**) on the defectiveness of CNTs. The inset in Figure 5c shows the dependence of the piezoelectric coefficient of CNTs on the full width at half maximum of the G-peak of the CNT spectra, reflecting the change in the curvature of the surface of the graphene sheet forming the CNT.

The analysis of the obtained results showed that with the increasing of the growth temperature from 615 to 690 °C, the effective piezoelectric coefficient of CNTs decreased from 0.281 to 0.028 C/m^2^ (Figure 7a). This dependence was due to the fact that with the increasing of their temperature, there was a decrease in the defectiveness of CNTs (Figure 3) and an increase in their diameter (Table 1). An increase in the thickness of the catalytic Ni layer at a fixed temperature of 675 °C also led to a change in the effective piezoelectric coefficient of the CNTs (Table 1, Figure 7b). The change in the effective piezoelectric coefficients of the CNTs, with an increase in the thickness of the catalytic layer, was primarily due to a change in the diameter of CNTs (Table 1), since at a temperature of 675 °C, a change in the thickness of the catalytic layer did not lead to a significant change in the defectiveness of the CNTs (Figure 3). With a simultaneous increase in the growth temperature and the thickness of the catalytic layer, a sharp decrease in the effective piezoelectric coefficient of CNTs was observed (Table 1).

This dependence was associated with a significant decrease in the defectiveness of the CNTs (Figure 3b) and an increase in the diameter of the CNTs (Table 1). The dependence of the effective piezoelectric coefficient of the CNTs on their defectiveness is shown in Figure 7c. It should be noted that a sharp increase in the effective piezoelectric coefficient of the CNTs was also observed with the increasing of the width at half maximum of the Raman G-peak of the CNT spectra, reflecting a decrease in the CNT diameter and an increase in the curvature of the graphene sheet forming it, respectively (inset in Figure 7c).

The CNT sample grown at the maximum temperature and thickness values of the catalytic layer (T = 690 °C and t = 30 nm) was knocked out of this dependence, with the value of the effective piezoelectric coefficient in that case being 0.297 C/m^2^. This deviation was probably due to the hierarchical structure of the CNT array and the presence of small-diameter CNTs on the tops of large-diameter CNTs (Figure 1).

Thus, it can be concluded that the effective piezoelectric coefficient of a CNT is determined by the structural perfection and diameter of the nanotubes. A decrease in the value of the piezoelectric coefficient of a CNT with the increasing of its diameter is explained by the decrease in the curvature of the surface of the graphene sheet, which is the main source of polarization due to the asymmetric redistribution of the electron density [22]. The obtained values correspond well with the previously calculated values of the piezoelectric coefficient of graphene [2,9,18,22].

## 4. Conclusions

We studied the structure and piezoelectric properties of multi-walled carbon nanotubes grown at different temperatures and thicknesses of the catalytic Ni layer. It was shown that carbon nanotubes grown using the PECVD method have a sufficiently high defectiveness due to the presence of bamboo-like structural defects and a catalytic center at the top of CNTs, which can be the source of their anomalous piezoelectric properties. It was shown that an increase in the growth temperature from 615 to 690 °C makes it possible to reduce the defectiveness of CNTs, which leads to a decrease in the effective piezoelectric coefficient of CNTs by more than 10 times. It was discovered that, with a similar degree of defectiveness, the value of the effective piezoelectric coefficient of a CNT is significantly affected by its diameter, which is explained by the increase in the curvature of the surface of the graphene sheet forming the CNT with the decreasing of the CNT diameter. This leads to an increase in polarization due to the asymmetric redistribution of the electron density [18]. The calculated values of the effective piezoelectric coefficient of CNTs range from 0.019 to 0.413 C/m^2^, depending on the degree of their defectiveness and diameter. The obtained values are comparable to or exceed the values of the piezoelectric coefficient of the deformed graphene [2,9,18,22]. In addition, the value of the piezoelectric coefficient of CNTs is comparable to the coefficient of a-quartz (0.171 C/m^2^) and the main nanoscale piezoelectric materials: BaTiO_3_ [13], ZnO [13], h-BN [50]. In this case, it is necessary to take into account that the magnitude of the effective piezoelectric coefficient of CNTs can increase due to the increase in deformation [18]. Therefore, in this work, the magnitude of the relative deformation is about 0.01%. However, the high values of Young’s modulus [51] and adhesive strength [52] of aligned carbon nanotubes make it possible to withstand a relative deformation of more than 10%. It was found that an increase in the thickness of the catalytic Ni layer leads not only to a decrease in the defectiveness of grown CNTs, but also to the branching and hierarchy of the CNT array. The hierarchical structure of CNTs causes an almost double increase in the effective piezoelectric coefficient in relation to a vertically aligned CNT, despite a decrease in the degree of its defectiveness being observed.

The obtained results suggest that defects are the cause of the anomalous piezoelectric effect in CNTs, which leads to the appearance of hysteresis in the current–voltage characteristics of deformed CNTs. These defects are structural and formed during growth during the application of the PECVD method. In this case, the concentration of defects and the diameter of CNTs can be controlled by changing the parameters of their growth. This also makes it possible to control the magnitude of the effective piezoelectric coefficient of CNTs. The presence of piezoelectric properties in carbon nanotubes is a new impetus for fundamental research on the piezoelectric properties in nanoscale materials in general and opens up a number of new possibilities for their application in the field of nanoelectromechanical device creation. In particular, the obtained results can be used to create highly efficient nanogenerators based on CNTs that are capable of converting external mechanical effects of the environment into electric current. In addition, the piezoelectric properties of carbon nanotubes make them one of the most promising materials for creating nano-piezotronic devices. Our future research will be dedicated to this area.

## Figures and Tables

**Table 1:** Calculated values of the effective piezoelectric coefficients of CNTs.

CNT Growth Parameters	CNT Diameter (D), nm	CNT Length (L), μm	Piezoelectric Coefficient (e), C/m^2^
Temperature at a catalytic layer thickness of 15 nm			
615 °C	47	31.2	0.281
630 °C	56	24.7	0.289
645 °C	59	24.2	0.127
660 °C	66	20.7	0.054
675 °C	58	16.9	0.05
690 °C	79	30.8	0.028
Catalytic layer thickness Ni at a temperature of 675 °C			
5 nm	62	11.3	0.021
10 nm	48	18.0	0.119
15 nm	58	16.9	0.05
20 nm	63	19.6	0.033
30 nm	64	10.7	0.037
Mix of catalytic layer thickness and temperature			
615 °C, 5 nm	32	8.3	0.413
630 °C, 10 nm	44	24.2	0.244
645 °C, 15 nm	59	24.2	0.127
660 °C, 20 nm	88	22.3	0.019
690 °C, 30 nm	92	40.5	0.297

## Data Availability

The data presented in this study are available on request from the corresponding author.

## Supplementary Materials

The following are available online at https://www.mdpi.com/article/10.3390/nano11112912/s1.

## References

[1] Maranganti R., Sharma N.D., Sharma P. (2006). Electromechanical coupling in nonpiezoelectric materials due to nanoscale nonlocal size effects: Green’s function solutions and embedded inclusions. Phys. Rev. B.

[2] Ong M.T., Reed E.J. (2012). Engineered Piezoelectricity in Graphene. ACS Nano.

[3] Sharma M., Srinivas V., Madras G., Bose S. (2016). Outstanding dielectric constant and piezoelectric coefficient in electrospun nanofiber mats of PVDF containing silver decorated multiwall carbon nanotubes: Assessing through piezoresponse force microscopy. RSC Adv..

[4] Sharma N., Maranganti R., Sharma P. (2007). On the possibility of piezoelectric nanocomposites without using piezoelectric materials. J. Mech. Phys. Solids.

[5] Blonsky M.N., Zhuang H.L., Singh A.K., Hennig R.G. (2015). Ab Initio Prediction of Piezoelectricity in Two-Dimensional Materials. ACS Nano.

[6] Zhu H., Wang Y., Xiao J., Liu M., Xiong S., Wong Z.J., Ye Z., Ye Y., Yin X., Zhang X. (2014). Observation of piezoelectricity in free-standing monolayer MoS2. Nat. Nanotechnol..

[7] Zelisko M., Hanlumyuang Y., Yang S., Liu Y., Lei C., Li J., Ajayan P.M., Sharma P. (2014). Anomalous piezoelectricity in two-dimensional graphene nitride nanosheets. Nat. Commun..

[8] Chu K., Yang C.-H. (2017). Nonlinear flexoelectricity in noncentrosymmetric crystals. Phys. Rev. B.

[9] Wang X., Tian H., Xie W., Shu Y., Mi W.-T., Mohammad M.A., Xie Q.-Y., Yang Y., Xu J.-B., Ren T.-L. (2015). Observation of a giant two-dimensional band-piezoelectric effect on biaxial-strained graphene. NPG Asia Mater..

[10] Hadjesfandiari A.R. (2013). Size-dependent piezoelectricity. Int. J. Solids Struct..

[11] He L., Lou J., Zhang A., Wu H., Du J., Wang J. (2017). On the coupling effects of piezoelectricity and flexoelectricity in piezoelectric nanostructures. AIP Adv..

[12] Zhang J., Wang C., Bowen C. (2014). Piezoelectric effects and electromechanical theories at the nanoscale. Nanoscale.

[13] Dai S., Gharbi M., Sharma P., Park H. (2011). Surface piezoelectricity: Size effects in nanostructures and the emergence of piezoelectricity in non-piezoelectric materials. J. Appl. Phys..

[14] Yudin P.V., Tagantsev A.K. (2013). Fundamentals of flexoelectricity in solids. Nanotechnology.

[15] El-Kelany K.E., Carbonniere P., Erba A., Sotiropoulos J.-M., Rérat M. (2016). Piezoelectricity of Functionalized Graphene: A Quantum-Mechanical Rationalization. J. Phys. Chem. C.

[16] Bistoni O., Barone P., Cappelluti E., Benfatto L., Mauri F. (2019). Giant effective charges and piezoelectricity in gapped graphene. 2D Mater..

[17] Duggen L., Willatzen M., Wang Z.L. (2018). Mechanically Bent Graphene as an Effective Piezoelectric Nanogenerator. J. Phys. Chem. C.

[18] Kundalwal S., Meguid S., Weng G. (2017). Strain gradient polarization in graphene. Carbon.

[19] Javvaji B., He B., Zhuang X. (2018). The generation of piezoelectricity and flexoelectricity in graphene by breaking the materials symmetries. Nanotechnology.

[20] Kundalwal S.I., Shingare K.B., Rathi A. (2019). Effect of flexoelectricity on the electromechanical response of graphene nano-composite beam. Int. J. Mech. Mater. Des..

[21] Rodrigues G.D.C., Zelenovskiy P., Romanyuk K., Luchkin S., Kopelevich Y., Kholkin A. (2015). Strong piezoelectricity in single-layer graphene deposited on SiO2 grating substrates. Nat. Commun..

[22] Chandratre S., Sharma P. (2012). Coaxing graphene to be piezoelectric. Appl. Phys. Lett..

[23] Ong M.T., Duerloo K.-A.N., Reed E.J. (2013). The Effect of Hydrogen and Fluorine Coadsorption on the Piezoelectric Properties of Graphene. J. Phys. Chem. C.

[24] Han J.K., Jeon D.H., Cho S.Y., Kang S.W., Yang S.A., Bu S.D., Myung S., Lim J., Choi M., Lee M. (2016). Nanogenerators consisting of direct-grown piezoelectrics on multi-walled carbon nanotubes using flexoelectric effects. Sci. Rep..

[25] Kvashnin A.G., Sorokin P.B., Yakobson B.I. (2015). Flexoelectricity in Carbon Nanostructures: Nanotubes, Fullerenes, and Nanocones. J. Phys. Chem. Lett..

[26] Yang Y., Tian H., Sun H., Xu R.-J., Shu Y., Ren T.-L. (2013). A spring-connected nanogenerator based on ZnO nanoparticles and a multiwall carbon nanotube. RSC Adv..

[27] Gao Y., Zhai Q., Barrett R., Dalal N.S., Kroto H.W., Acquah S.F.A. (2013). Piezoelectric enhanced cross-linked multi-walled carbon nanotube paper. Carbon.

[28] Han J.K., Lim J., Jeon D.H., Cho S.Y., Kang S.W., Bu S.D. (2017). Flexible Piezoelectric Generators by Using the Bending Motion Method of Direct-Grown-PZT Nanoparticles on Carbon Nanotubes. Nanomaterials.

[29] Il’Ina M.V., Il’In O.I., Blinov Y.F., Smirnov V.A., Kolomiytsev A.S., Fedotov A., Konoplev B.G., Ageev O.A. (2017). Memristive switching mechanism of vertically aligned carbon nanotubes. Carbon.

[30] Il’Ina M., Il’In O., Rudyk N., Konshin A., Ageev O. (2018). The memristive behavior of non-uniform strained carbon nanotubes. Nanosyst. Physics, Chem. Math..

[31] Il’Ina M.V., Il’In O.I., Guryanov A.V., Osotova O.I., Blinov Y.F., Fedotov A.A., Ageev O.A. (2021). Anomalous piezoelectricity and conductivity in aligned carbon nanotubes. J. Mater. Chem. C.

[32] Il’Ina M.V., Il’In O.I., Blinov Y.F., Konshin A.A., Konoplev B.G., Ageev O.A. (2018). Piezoelectric Response of Multi-Walled Carbon Nanotubes. Materials.

[33] Ouyang Y., Cong L., Chen L., Liu Q., Fang Y. (2008). Raman study on single-walled carbon nanotubes and multi-walled carbon nanotubes with different laser excitation energies. Phys. E Low-Dimens. Syst. Nanostruct..

[34] Mathur A., Tweedie M., Roy S.S., Maguire P.D., McLaughlin J.A. (2009). Electrical and Raman spectroscopic studies of vertically aligned multi-walled carbon nanotubes. J. Nanosci. Nanotechnol..

[35] Dresselhaus M.S., Jorio A., Hofmann M., Dresselhaus G., Saito R. (2010). Perspectives on Carbon Nanotubes and Graphene Raman Spectroscopy. Nano Lett..

[36] Kuznetsov V.L., Bokova-Sirosh S., Moseenkov S.I., Ishchenko A.V., Krasnikov D.V., Kazakova M.A., Romanenko A., Tkachev E.N., Obraztsova E. (2014). Raman spectra for characterization of defective CVD multi-walled carbon nanotubes. Phys. Status Solidi (B).

[37] Brown S.D.M., Jorio A., Corio P., Dresselhaus M.S., Dresselhaus G., Saito R., Kneipp K. (2001). Origin of the Breit-Wigner-Fano lineshape of the tangentialG-band feature of metallic carbon nanotubes. Phys. Rev. B.

[38] Jiang J.-W., Wang J.-S. (2010). Conditions for the existence of phonon localized edge-modes. Phys. Rev. B.

[39] Il’Ina M.V., Il’In O.I., Blinov Y.F., Smirnov V.A., Ageev O.A. (2018). Nonuniform Elastic Strain and Memristive Effect in Aligned Carbon Nanotubes. Tech. Phys..

[40] Ageev O.A., Blinov Y.F., Il’ina M.V., Konoplev B.G., Smirnov V.A., Tiwari A., Mishra Y.K., Kobayashi H., Turner A.P.F. (2017). Resistive Switching of Vertically Aligned Carbon Nanotubes for Advanced Nanoelectronic Devices. Intelligent Nanomaterials.

[41] Il’Ina M.V., Il’In O.I., Guryanov A.V., Osotova O.I., Ageev O.A. (2019). Dependence of the memristor effect of carbon nanotube bundles on the pressing force. Full-Nanotub. Carbon Nanostruct..

[42] Lu W., Wang D., Chen L. (2007). Near-Static Dielectric Polarization of Individual Carbon Nanotubes. Nano Lett..

[43] Obitayo W., Liu T. (2012). A Review: Carbon Nanotube-Based Piezoresistive Strain Sensors. J. Sensors.

[44] Cao J., Wang Q., Dai H. (2003). Electromechanical Properties of Metallic, Quasimetallic, and Semiconducting Carbon Nanotubes under Stretching. Phys. Rev. Lett..

[45] Kuzumaki T., Mitsuda Y. (2004). Dynamic measurement of electrical conductivity of carbon nanotubes during mechanical deformation by nanoprobe manipulation in transmission electron microscopy. Appl. Phys. Lett..

[46] Liu B., Jiang H., Johnson H., Huang Y. (2004). The influence of mechanical deformation on the electrical properties of single wall carbon nanotubes. J. Mech. Phys. Solids.

[47] Tombler T.W., Zhou C., Alexseyev L., Kong J., Dai H., Liu L., Jayanthi C.S., Tang M., Wu S.-Y. (2000). Reversible electromechanical characteristics of carbon nanotubes underlocal-probe manipulation. Nature.

[48] Mohamed N.M., Kou L.M. (2011). Piezoresistive Effect of Aligned Multiwalled Carbon Nanotubes Array. J. Appl. Sci..

[49] Il’Ina M.V., Il’In O.I., Smirnov V.A., Blinov Y.F., Konoplev B.G., Ageev O.A. (2018). Scanning Probe Techniques for Characterization of Vertically Aligned Carbon Nanotubes. At. -Force Microsc. Its Appl..

[50] Nakhmanson S.M., Calzolari A., Meunier V., Bernholc J., Nardelli M.B. (2003). Spontaneous polarization and piezoelectricity in boron nitride nanotubes. Phys. Rev. B.

[51] Ageev O., Il’In O.I., Kolomiitsev A.S., Konoplev B.G., Rubashkina M.V., Smirnov V.A., Fedotov A. (2012). Development of a technique for determining Young’s modulus of vertically aligned carbon nanotubes using the nanoindentation method. Nanotechnologies Russ..

[52] Ageev O.A., Blinov Y.F., Il’Ina M.V., Il’In O.I., Smirnov V.A., Tsukanova O.G. (2016). Study of adhesion of vertically aligned carbon nanotubes to a substrate by atomic-force microscopy. Phys. Solid State.

