# Supplementary material for: Analysis of the Piezoelectric Properties of Aligned Multi-Walled Carbon Nanotubes
Source: Nanomaterials (Basel). 2021 Oct 30;11(11):2912. doi: 10.3390/nano11112912 (PMC8617926; doi:10.3390/nano11112912)
Supplement: Supplementary file 1 [file nanomaterials-11-02912-s001.zip › nanomaterials-1392194-supplementary.pdf]

nanomaterials

MDPI

Analysis of the Piezoelectric Properties of Aligned Multi-Walled Carbon Nanotubes

Marina V. Il'ina [1,*], Oleg I. Il'in [2], Nikolay N. Rudyk [2], Olga I. Osotova [2] and Alexander A. Fedotov [1] and Oleg A. Ageev [1,3]

[1] Institute of Nanotechnologies, Electronics and Electronic Equipment Engineering, Southern Federal University, 347922 Taganrog, Russia; aafedotov@sfedu.ru (A.A.F.); ageev@sfedu.ru (O.A.A.)
[2] Research Laboratory of Functional Nanomaterials Technology, Southern Federal University, 347922 Taganrog, Russia; oiilin@sfedu.ru (O.I.I.); nnrudyk@sfedu.ru (N.N.R.); osotova@sfedu.ru (O.I.O.)
[3] Research and Education Center "Nanotechnologies", Southern Federal University, Taganrog 347922, Russia
* Correspondence: mailina@sfedu.ru

As we noted before, the occurrence of an elastic strain ΔL in the carbon nanotube will lead to the formation of an internal field strength E_{def} associated with the manifestation of

piezoelectric effect. According to the theory of the piezoelectric effect, the internal field value E_{def} can be estimated as:

$$E_{def} = \frac{e}{\varepsilon_0 \varepsilon_{||}} \frac{\Delta L}{L} \quad (1)$$

where $\varepsilon_{||}$ is the longitudinal dielectric constant of a CNT; $e = \sigma/E$ is the piezoelectric coefficient, and σ is the mechanical stress of the CNT. Under the condition of the formation of tensile deformation, the positive piezoelectric charges are focused on the nanotube top and an internal field strength E_{def} is directed from the top to the bottom of the nanotube.

When a sawtooth voltage pulse is applied to a strained CNT, a redistribution of deformation ΔL and the corresponding internal electric field E_{def} is observed under the action of the inverse piezoelectric effect. In the region of the current–voltage characteristic (CVC) from −3 to 3 V, an additional compressive strain arises ($\Delta L(t) = \Delta L - \Delta L'(t)$) and the value of the corresponding initial internal field $E_{def}(t)$ decreases. As a result, the resistance of the nanotube decreases and corresponds to the low-resistance state R_{LR}.

At the time $t = t_0$ ($U = 3$ V), there is a jump of dU/dt and the relaxation of excessive stress. As a result, there is a hysteresis, the area of which is determined by the value of the oppositely directed current density [29]:

$$i(t_0) = (E(t_0) + E_{def}(t_0)) \frac{L\varepsilon_0\varepsilon_{||}}{U(t_0)} \frac{dE(t)}{dt} - \frac{\Delta L(t)}{Upiezo} \frac{d\sigma(t)}{dt} \quad (2)$$

where $Upiezo$ is the potential corresponding to the strain ΔL. As a result, the nanotube switches into a high-resistance state, with resistance $R(t)$ being proportional to the strain $\Delta L(t) = \Delta L + \Delta L'(t_0)$.

In region from 3 to −3 V of the CVC, the internal field $E_{def}(t)$ increases due to additional strain $\Delta L(t) = \Delta L + \Delta L'(t)$ and partially compensates for the external field $E(t)$. At $U(t) \rightarrow 0$, the strain $\Delta L'(t)$ tends to zero and the resistance is determined by the initial strain ΔL of the nanotube.

Thus, the redistribution of strain and piezoelectric charge leads to hysteresis in the current–voltage characteristics and the appearance of memristive switching of the strained carbon nanotube.

Citation: Il'ina, M.V.; Il'in, O.I.; Rudyk, N.N.; Osotova, O.I.; Fedotov, A.A. Analysis of the Piezoelectric Properties of Aligned Multi-Walled Carbon Nanotubes. *Nanomaterials* **2021**, *11*, 2912. https://doi.org/10.3390/nano11112912

Academic Editor: Marianna V. Kharlamova

Received: 7 September 2021
Accepted: 26 October 2021
Published: 30 October 2021

Publisher's Note: MDPI stays neutral with regard to jurisdictional claims in published maps and institutional affiliations.

Copyright: © 2021 by the authors. Licensee MDPI, Basel, Switzerland. This article is an open access article distributed under the terms and conditions of the Creative Commons Attribution (CC BY) license (http://creativecommons.org/licenses/by/4.0/).
